# Combined deletion of *Xrcc4* and *Trp53* in mouse germinal center B cells leads to novel B cell lymphomas with clonal heterogeneity

**DOI:** 10.1186/s13045-015-0230-5

**Published:** 2016-01-07

**Authors:** Zhangguo Chen, Mihret T. Elos, Sawanee S. Viboolsittiseri, Katherine Gowan, Sonia M. Leach, Michael Rice, Maxwell D. Eder, Kenneth Jones, Jing H. Wang

**Affiliations:** Department of Immunology and Microbiology, University of Colorado, Anschutz Medical Campus, 12800 E. 19th Ave, Mail Stop 8333, Aurora, CO 80045 USA; Department of Biomedical Research, National Jewish Health, Denver, CO 80206 USA; Integrated Center for Genes, Environment and Health, National Jewish Health, Denver, CO 80206 USA; Department of Biochemistry and Molecular Genetics, University of Colorado, Anschutz Medical Campus, Aurora, CO 80045 USA

**Keywords:** Non-homologous end-joining, Genomic instability, Chromosomal translocations, B cell lymphoma, Clonal heterogeneity

## Abstract

**Background:**

Activated B lymphocytes harbor programmed DNA double-strand breaks (DSBs) initiated by activation-induced deaminase (AID) and repaired by non-homologous end-joining (NHEJ). While it has been proposed that these DSBs during secondary antibody gene diversification are the primary source of chromosomal translocations in germinal center (GC)-derived B cell lymphomas, this point has not been directly addressed due to the lack of proper mouse models.

**Methods:**

In the current study, we establish a unique mouse model by specifically deleting a NHEJ gene, *Xrcc4*, and a cell cycle checkpoint gene, *Trp53*, in GC B cells, which results in the spontaneous development of B cell lymphomas that possess features of GC B cells.

**Results:**

We show that these NHEJ deficient lymphomas harbor translocations frequently targeting immunoglobulin (Ig) loci. Furthermore, we found that *Ig* translocations were associated with distinct mechanisms, probably caused by AID- or RAG-induced DSBs. Intriguingly, the AID-associated *Ig* loci translocations target either *c-myc* or *Pvt-1* locus whereas the partners of RAG-associated *Ig* translocations scattered randomly in the genome. Lastly, these NHEJ deficient lymphomas harbor complicated genomes including segmental translocations and exhibit a high level of ongoing DNA damage and clonal heterogeneity.

**Conclusions:**

We propose that combined NHEJ and p53 defects may serve as an underlying mechanism for a high level of genomic complexity and clonal heterogeneity in cancers.

**Electronic supplementary material:**

The online version of this article (doi:10.1186/s13045-015-0230-5) contains supplementary material, which is available to authorized users.

## Background

The occurrence of human B cell lymphomas is much more frequent than that of T cell lymphomas [1]. This phenomenon might be attributed to the multiple mechanisms functioning in B lymphocytes that intrinsically generate DNA double-stranded breaks (DSBs) or mutations [2, 3]. Developing B cells in the bone marrow (BM) undergo V(D)J recombination to assemble the variable (V) region exons of *Ig* genes [4, 5]. V(D)J recombination involves a cut-and-join mechanism initiated by the lymphocyte-specific RAG1/2 endonucleases that recognize and introduce DSBs at recombination signal sequences (RSS) flanking germline V, D, and J segments [6]. Subsequently, broken V, D, and J segments are joined by ubiquitous non-homologous end-joining (NHEJ) [7]. Ongoing RAG-expression in newly generated B cells allows secondary V(D)J recombination, termed “receptor editing”, a process in which additional *Ig* gene rearrangements may occur in BM immature B cells [8–12]. Ultimately, RAG down-regulation in mature B cells prohibits further V(D)J rearrangement [13, 14]. However, our previous studies suggest that mature B cells may also undergo secondary V(D)J recombination at low frequency in an in vitro culture system [15]. While RAG contributes to the genomic instability of developing B cells [16–18], its role in mature B cell lymphomagenesis is still under debate.

Upon antigen activation, mature B cells undergo further genetic diversification processes, namely, class switch recombination (CSR) and somatic hypermutation (SHM), in specialized secondary lymphoid structures termed germinal centers (GCs) [19–22]. Activation-induced deaminase (AID) initiates CSR and SHM [23, 24], which deaminates cytosines in transcribed DNA and ultimately causes DSBs or point mutations [25–28]. CSR is a region-specific deletional recombination process required for producing isotype-switched antibody such as IgG [29]. AID-initiated DSBs occur at the switch (S) regions within the *Igh* locus, which are eventually resolved as deletions *in cis* on the same chromosomes, thereby causing the switch of constant regions of *Igh* [29]. SHM introduces predominantly point mutations into IgH and IgL V region exons, allowing the selection of B cell clones with increased affinity for antigen [27]. Besides *Ig* loci, AID can target non-Ig loci to induce genetic lesions, thereby posing a threat to genome stability [30]. Consistently, the dysregulated AID activity contributes to tumorigenesis [31, 32]. We and others have shown that AID is required for generating chromosomal breaks at the *Igh* locus [15] and the *Igh-c-myc* translocations [33].

Apart from programmed DSBs, B lymphocytes harbor general DSBs arising from genotoxic agents such as oxidative damage or DNA replication errors. To preserve genome integrity, two major DSB repair pathways operate in mammalian cells: homologous recombination (HR) and NHEJ. While HR-directed repair requires homologous templates, NHEJ can repair DSBs with little or no sequence homology [34]. The NHEJ pathway joins programmed DSBs in lymphocytes including RAG- or AID-initiated DSBs [35] and repairs general DSBs in all types of cells [34]. The NHEJ pathway includes Ku70, Ku80, DNA-PKcs, XLF, Artemis, XRCC4, and DNA Ligase 4 (Lig4) [34]. XRCC4, Lig4, and possibly XLF form a complex to catalyze the end-ligation step of NHEJ [34, 36]. Germline deletion of NHEJ results in severe combined immune deficiency due to inability to complete V(D)J recombination [4, 7]. Conditional deletion of *Xrcc4* or *Lig4* in peripheral B cells reduces the CSR level and causes a high level of chromosomal breaks and translocations at the *Igh* locus due to inability to repair AID-initiated DSBs [15, 37]. While defective DSB repair leads to genomic instability, cell cycle checkpoints can protect organisms from adverse downstream effects, such as transformation, by eliminating damaged cells. As DSB repair and checkpoint mechanisms complement each other, loss of both can cause dramatic predisposition to transformation in mouse lymphocytes, often leading to lymphomas due to the inappropriate repair of programmed or general DSBs [38]. For instance, deficiency of *Xrcc4*, *Lig4*, and *Xrcc6* (*Ku70)* in conjunction with *Trp53* deficiency causes pro-B cell lymphomas carrying co-amplified *Igh-c-myc* loci [39–43]. *TP53* is a well-known tumor suppressor gene, which encodes p53 protein capable of responding to diverse cellular stresses by regulating the expression of its target genes, thereby inducing cell cycle arrest, apoptosis, or senescence, modulating DNA repair or metabolism and serving as the guardian of the genome [44–46].

We previously showed that conditionally deleting *Xrcc4* in *Trp53*-deficient peripheral B cells resulted in the development of surface Ig negative lymphomas from editing and switching B cells (termed CXP lymphomas) [47]. Although CXP tumors have mature B cell characteristics, they appear to be very different from human mature B cell lymphomas. For instance, CXP lymphomas do not express IgH or IgL chain protein on the surface or intracellularly and show no SHM in the rearranged VDJ exon [47]. In contrast, most of human mature B cell lymphomas are surface Ig positive except classical Hodgkin’s lymphoma and a few others [1]. These differences suggest that the mechanism of lymphomagenesis and the developmental stage of tumor progenitors are very different between CXP and human mature B cell lymphomas. Such difference may be due to the relatively early deletion of *Xrcc4* via CD21cre. CD21 begins to be expressed between the immature and the mature B cell stages, specifically in transitional B cells [48]. Thus, in mice performing CD21cre-mediated *Xrcc4* deletion, it is likely that some DSBs are generated before the cells are recruited into the GC reaction. In the current study, we delete *Xrcc4* and *Trp53* at a later stage of mature B cell development during the GC reaction, which leads to B cell lymphomas that possess GC B cell features and harbor frequent *Ig* loci translocations, ongoing DNA damage and a high level of clonal heterogeneity.

## Results

### Deletion of *Xrcc4* but not *Trp53* via Cγ1cre leads to a high level of genomic instability at the *Igh* locus

**Fig. 1:**
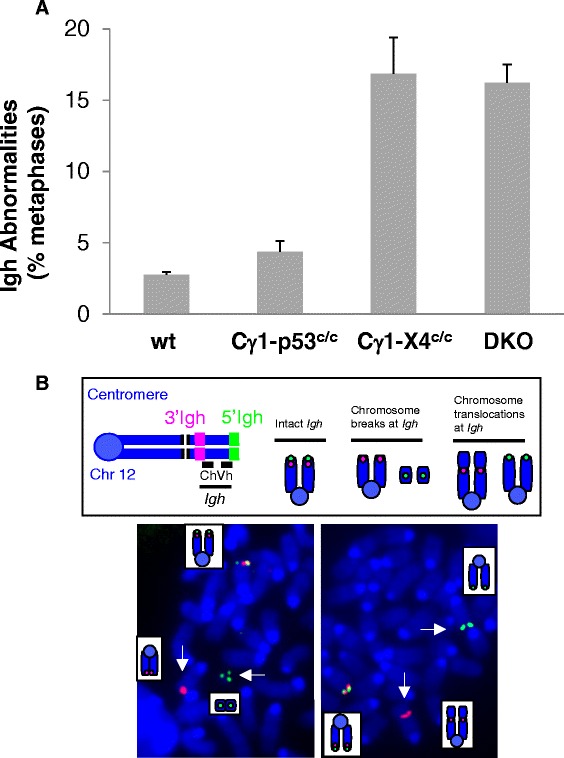
Deletion of *Xrcc4* but not *Trp53* via Cγ1cre leads to a high level of genomic instability at the *Igh* locus. **a** Quantification of *Igh* abnormalities in day 4 anti-CD40/IL4-activated wt (*n* = 6), Cγ1-p53^c/c^ (*n* = 4), Cγ1-X4^c/c^ (*n* = 3), and *DKO* (*n* = 6) splenic B cells. Data are presented as mean ± s.e.m. Statistical analyses were calculated by a Student’s *t* test with two-tailed distribution and equal variance. *p* = 0.024 (control vs Cγ1-p53^c/c^), *p* = 6.78E-05 (control vs Cγ1-X4^c/c^), *p* = 1.03E-06 (control vs DKO), *p* = 0.805 (Cγ1-X4^c/c^ vs DKO). **b** *Top*: Diagram of *Igh* metaphase FISH probes and abnormalities. An intact *Igh* shows co-localized *red* and *green* signals while a broken locus appears as split *red* and *green* signals. *Bottom*: Example of metaphase FISH showing *Igh* breaks indicated with *white arrows*

We established a new mouse model in which *Xrcc4* (*X*) and *Trp53* (*P*) genes were deleted at a later stage of mature B cell development using Cγ1Cre [49]. In Cγ1Cre knock-in mice, Cre expression is driven by the endogenous Iγ1 promoter and occurs in the majority of GC B cells [49]. Consistently, we found that GC B cells have the complete deletion of *Xrcc4* (*X*) floxed allele in *Cγ1Cre/X*^*c/+*^ mice (c: floxed allele; +: wt allele) (Additional file 1: Figure S1A). Then, we examined whether deletion of *Xrcc4* or *Trp53* via Cγ1cre led to a high level of genomic instability in primary B cells. To do so, we assayed for *Igh* locus-specific genomic instability in B cells that can be induced via anti-CD40/IL4 activation in the in vitro culture of primary B cells [15, 37]. Using metaphase fluorescence in situ hybridization (FISH), we found that anti-CD40/IL4-stimulated *Xrcc4/Trp53*-double deficient (DKO) B cells harbored a high level of *Igh* locus instabilities, including breaks and translocations (Fig. 1a, b). Furthermore, our data showed that *Xrcc4* deficiency alone resulted in a high level of *Igh* locus genomic instability; in contrast, *Trp53* deficiency did not enhance the level of genomic instability in activated B cells compared to wt controls (Fig. 1a). Thus, the *Igh* locus genomic instability in the DKO B cells is largely attributable to *Xrcc4* deficiency.

### Combined deletion of *Xrcc4* and *Trp53* via Cγ1cre predisposes B cells to lymphomagenesis

**Fig. 2:**
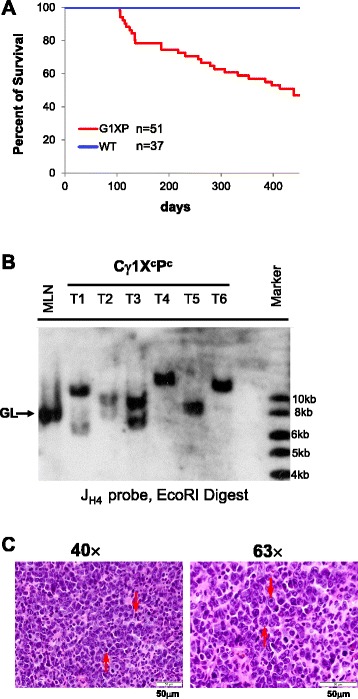
Establishment of the G1XP B cell lymphoma model. **a** Kaplan-Meier survival curve: percent survival of wt controls (*n* = 37) and *Cγ1CreX* ^*c*^ *P* ^*c*^ mice (*n* = 51) vs age in days is shown. **b** Southern Blot analysis of second cohort of *Cγ1CreX* ^*c*^ *P* ^*c*^ mice for J_H_ rearrangements. G1XP lymphoma genomic DNA was employed for EcoRI digestion and hybridized with the J_H4_ probe. Germline (GL) bands are indicated (*arrow heads*). MLN stands for mesenteric lymph node. **c** H&E analysis of G1XP lymphomas. *Left*: a high level of apoptotic cells (*red arrows*) (objective ×40); *Right*: diffusely enlarged nuclei (*red arrow*, *bottom*) and salient nucleoli (*red arrow*, *top*) (objective ×63). Scale bars 50 μm

We conducted cohort studies by monitoring two cohorts of *Cγ1CreX*^*c*^*P*^*c*^ mice for the occurrence of B cell lymphomas (*X*^*c*^: *X*^*c/c*^ or *X*^*c/−*^; *P*^*c*^: *P*^*c/c*^ or *P*^*c/−*^). Within the first cohort, we found that *Cγ1CreX*^*c*^*P*^*c/+*^ mice (Additional file 1: Figure S1B, *n* = 19, blue line) survived normally and were not cancer-prone. This phenotype is similar to that of *CD21CreX*^*c/-*^ mice [47] in that both lines only have *Xrcc4*, but not *Trp53*, deleted. *Cγ1CreX*^*c/+*^*P*^*c*^ mice (Additional file 1: Figure S1B, *n* = 20, green line), which still express XRCC4, developed thymic lymphomas or solid tumors. This phenotype is likely due to p53 haploinsufficiency as the genotypes of all the tumors are *P*^*c/−*^. In contrast, 11 out of 47 *Cγ1CreX*^*c*^*P*^*c*^ mice succumbed to B lineage lymphoma (Additional file 1: Figure S1B, *n* = 47, red line) as all 11 tumors expressed B lineage markers including B220 and CD19, while the rest of morbid mice died of thymic lymphomas or solid tumors. The floxed *Xrcc4* allele was deleted in all of the B lineage lymphomas but not in thymic lymphomas and solid tumors (data not shown). Within the second cohort, we observed a survival curve closely resembling the first one, with a similar penetrance and latency for tumor development (Fig. 2a), albeit the second cohort included only wt controls (*n* = 37) and *Cγ1CreX*^*c*^*P*^*c*^ mice (*n* = 51). We termed the *Cγ1CreX*^*c*^*P*^*c*^-derived B lineage tumors G1XP lymphomas.

### Phenotypic characterization of G1XP lymphomas

We performed Southern blotting to assay G1XP tumor DNA for *Igh* rearrangements and found that 7 out of 11 analyzable primary G1XP tumors from the first cohort exhibited clonal *Igh* rearrangements, along with varying degrees of a germline J_H_ band (Additional file 1: Figure S1C). However, the germline band usually occurred in low levels indicating derivation from non-B lineage cells within the tumor. Consistently, the tumor samples analyzed from the second cohort also displayed clonal rearrangements of the J_H_ allele (Fig. 2b). Histological analysis of the tumor samples showed that the lymphoma cells possessed diffusely enlarged cytoplasm, salient nuclei, and nucleoli (Fig. 2c), consistent with immunoblastic B cells. Phenotypic characterization of G1XP lymphomas was performed by flow cytometry (Additional file 1: Table S1). We found that all of the lymphoma samples consistently expressed B220, CD24, CD38, CD43, CD93, and CD138. The majority of lymphomas also expressed PNA, a marker for GC B cells (see below), and about 40 % of them were surface IgG positive (Additional file 1: Table S1).

To test whether G1XP lymphomas derived from B cells that had undergone SHM/CSR, we cloned the VDJ_H_ exons from three G1XP lymphomas using a PCR approach [47]. We found that G1XP tumors contained structurally normal in-frame or out-of-frame V(D)J rearrangements; more importantly, we sequenced these VDJ_H_ exons and downstream J_H_ introns and found that these three tumors harbored 13, 1, and 3 mutations, respectively, in the J_H_ regions (Additional file 1: Figure S2). These results further confirm that inactivation of *Xrcc4* and *Trp53* via Cγ1cre leads to B cell lymphomas capable of undergoing SHM. Thus, we conclude that the deletion of both *Xrcc4* and *Trp53* genes via Cγ1Cre results in the development of novel B cell lymphomas.

### Analysis of *Ig* loci translocation junctions identifies known and novel translocation partners

**Fig. 3:**
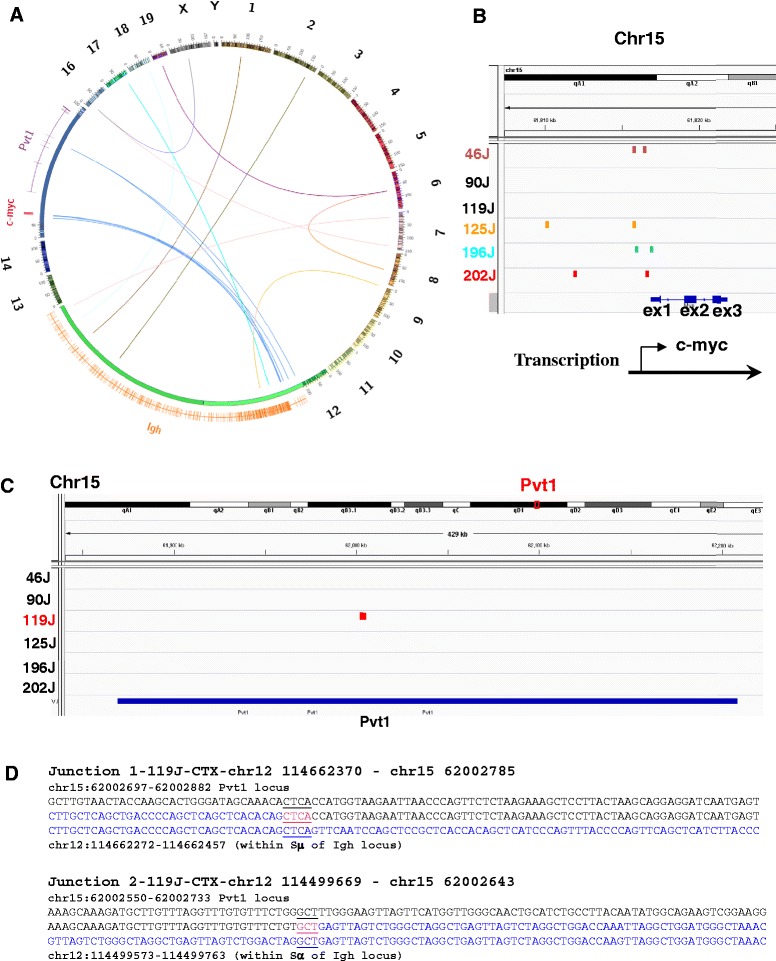
Analysis of CTX junctions identifies known and novel translocation partners. **a** A Circos plot depicts all CTXs involving *Igh*, *Igκ*, and *Igλ* loci. Individual chromosomes are shown as color-coded bars with specific banding patterns. The region on chr12 (in *green*) (114,480,000–117,249,000) is zoomed in ×400 including the *Igh* locus while the region on chr15 (in *blue*) (61,800,000–62,083,000) is zoomed in ×1600 including *c-myc* and *Pvt1* loci. All *Igh* CTXs involving S or C regions are translocated to *c-myc* or *Pvt1* locus shown as a cluster of *blue lines*. All other Ig CTXs involving V gene segments are translocated to various chromosomes shown as *different color-coded lines*. **b** The view within the IGV browser shows all the CTX breakpoints occurring upstream of or within *c-myc* 1st exon. **c** The location of *Pvt1* translocations shown in the IGV browser. **d** Analysis of junction sequences of the *Igh-Pvt1* translocation. Sequences from NGS are aligned with genomic sequences of mm9 with chr15 sequence in *black* and chr12 sequence in *blue*. Micro-homology at the junctions is in *red* and *underlined*

We performed whole genome next generation sequencing (NGS) to identify the inter-chromosomal translocations (CTX) involving *Ig* loci in 6 G1XP lymphomas. NGS allows us to molecularly characterize the CTX junctions at the base pair (bp) level, which may provide insights into the molecular mechanisms leading to these translocations. NGS identified 25 CTXs involving *Ig* genes, 20 of which were confirmed by manual alignment to reference databases (mm9), while the remainder was excluded due to alignment artifacts or sequencing limitations. Among the 20 CTXs, 16 of them occurred at the *Igh* locus, with 10 breakpoints present within or around S regions on chromosome 12q13.2 (Fig. 3a, Additional file 1: Table S2), indicating that the translocations originated from CSR process; the other 6 CTXs occurred in close proximity to various V_H_ gene segments of the *Igh* locus (Fig. 3a, Additional file 1: Table S2), suggesting that these CTXs were mediated by RAGs. The additional 4 CTXs targeted *Igκ* or *Igλ* loci on chromosome 6 or 16, respectively, with the breakpoints occurring in close proximity to Vλ or Vκ gene segments, consistent with RAG-mediated DSBs (Fig. 3a, Additional file 1: Table S2).

Notably, an interesting pattern emerged among these CTXs that apparently separated into two categories. First, all of the 10 CTXs occurring around S or C regions of the *Igh* locus were translocated to the *c-myc* or plasmacytoma variant translocation 1 (*Pvt1*) locus on chromosome 15 (8 vs 2, respectively) (Fig. 3a). The translocation breakpoints at the *c-myc* locus clustered in the 5′ non-coding regions on chromosome 15qD1 (Fig. 3b, Additional file 1: Table S2). Second, all of the 10 CTXs occurring close to V gene segments of *Igh*, *Igκ*, and *Igλ* were translocated to various chromosomes involving random genetic or intergenic regions (Fig. 3a) (Additional file 1: Table S2). This intriguing pattern of translocation partners suggests that distinct mechanisms operate to mediate these two categories of translocations, probably involving AID vs RAGs, respectively. NGS data revealed a novel translocation partner of *Igh* locus, which targeted the *Pvt1* locus (Fig. 3c), located about 30 kb telomeric of the *c-myc* locus. The *Pvt1* locus contains a long non-coding RNA gene, which is a frequent translocation partner in mouse plasmacytomas and variants of human Burkitt’s lymphomas (BL) [50, 51]. NGS data identified *Igh* breakpoints located within Sμ or Sα regions, strongly implicating the involvement of AID (Fig. 3d). The junctional sequences harbored micro-homology (MH), an indicator of alternative end-joining (A-EJ) [35, 52]. Taken together, our data suggest that *Ig* loci translocations are likely caused by AID or RAG activity.

Apart from CTX junctions, we also identified V(D)J recombination junctions from our NGS data. Since *Xrcc4* was deleted in the GC B cells, these lymphoma cells harbored normal D-J or V-D-J recombination junctions at the *Igh* locus (Additional file 1: Figure S3, junction 1 and 2, respectively). In contrast, we detected aberrant *Igλ* locus V-J rearrangements, which harbored large deletions of Vλ1 or Jλ3 exon including 41 bp of Vλ1 exon and the entire Jλ3 exon (Additional file 1: Figure S3, junction 3). These data are consistent with our previous results showing that CD21cre-mediated *Xrcc4* deletion led to aberrant *Igλ* rearrangements [15, 47]. We identified MH at the V-J junction (Additional file 1: Figure S3, junction 3), consistent with the involvement of A-EJ. Taken together, our data suggest that these aberrant *Igλ* rearrangements occurred in the absence of *Xrcc4*, probably in the context of secondary V(D)J recombination.

### Validation of clonal translocations involving *Ig* loci in G1XP lymphomas

**Fig. 4:**
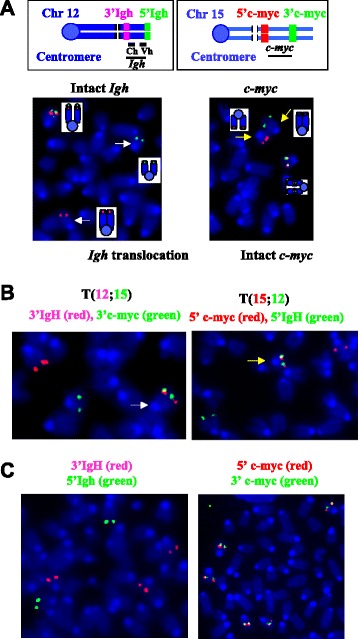
FISH validation of *Igh* locus translocations. **a**, **b** Inactivation of *Xrcc4* and *Trp53* via Cγ1-cre leads to reciprocal *Igh-c-myc* translocation in G1XP lymphoma (46 J). **a** *Upper left*: Diagram of *Igh* FISH probes. *Bottom left*: An example of metaphase *Igh* FISH image. An intact *Igh* shows co-localized *red* and *green* signals while a translocated locus appears as split *red* and *green* signals on different chromosomes (*white arrow*). *Upper right*: Diagram of *c-myc* FISH probes. *Bottom right*: An example of metaphase FISH showing *c-myc* locus translocation (*yellow arrow*) and an intact *c-myc* locus. **b** *Left*: A metaphase FISH image showing the t(12;15) translocation with 3′Igh (*red*) and 3′c-myc (*green*) probes juxtaposed on the derivative chr12 (*white arrow*). *Right*: A metaphase FISH image showing the t(15;12) translocation with 5′c-myc (*red*) and 5′Igh (*green*) probes juxtaposed on the derivative chr15 (*yellow arrow*). A normal chr12 and chr15 are also present in the metaphase. **c** *Left*: a metaphase FISH image showing the *Igh* locus translocations in G1XP lymphoma (119 J) with 3′ and 5′*Igh* probes split. *Right*: a metaphase FISH image showing the intact *c-myc* locus with 3′ and 5′ probes co-localized in the same lymphoma sample

To further validate the observed genomic instability, we performed metaphase FISH assay to detect locus-specific clonal translocations, which were also validated via independent methods (Additional file 1: Table S3). Of note, all of the detailed analysis utilized the tumor samples from the second cohort of *Cγ1CreX*^*c*^*P*^*c*^ mice. Among the six tumors sequenced by NGS, we confirmed that four out of six samples harbored clonal *c-myc* translocations while five out of six had *Igh* locus translocation (Fig. 4a). Clonal translocations were defined as more than 80 % of tumor metaphases harboring such translocations. Furthermore, we found that six out of nine analyzed G1XP lymphoma samples harbored clonal *c-myc* translocations while seven out of nine contained clonal *Igh* translocations. Next, we performed FISH analysis using 3′Igh (red) and 3′c-myc (green) probes or 5′c-myc (red) and 5′Igh probes (green) to determine whether these are reciprocal translocations occurring between *Igh* and *c-myc* loci. Indeed, we found that *Igh* and *c-myc* probes were juxtaposed on the translocated chromosomes (Fig. 4b). Thus, we conclude that the majority of G1XP lymphomas harbor *Igh-c-myc* reciprocal translocations. In addition to these translocations, we detected clonal translocations involving the *Igλ* locus (Additional file 1: Figure S4), one of the *Ig* light chain gene loci, in the majority of G1XP lymphomas analyzed.

**Fig. 5:**
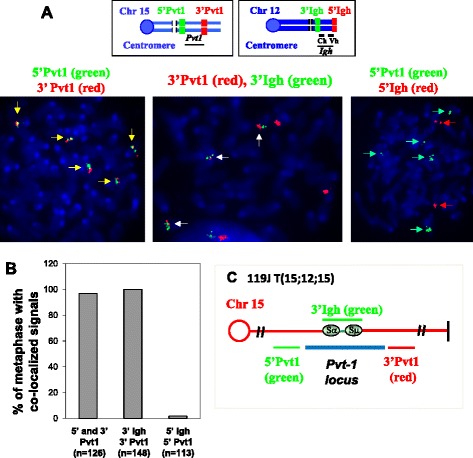
G1XP lymphomas harbor segmental translocations involving *Pvt1* locus. **a** Metaphase FISH analysis of the *Igh-Pvt1* translocation in G1XP lymphoma (119 J). *Top*: Diagrams of *Igh* or *Pvt1* FISH probes. *Bottom left*: five copies of the 5′ (*green*) and 3′ (*red*) *Pvt1* probes are co-localized (*yellow arrows*); *Bottom middle*: three copies of the 3′*Igh* (*green*) and 3′*Pvt1* probes are co-localized (*white arrows*); *Bottom right*: five copies of 5′*Pvt1* probe (*green*) and two copies of 5′ *Igh* probe (*red*) are not co-localized (indicated by *green* or *red arrows*, respectively). **b** Quantification of *Igh-Pvt1* translocations analysis in G1XP lymphoma (119 J). The number of analyzed metaphases is indicated. **c** Schematic map of t(15;12;15) translocation in G1XP 119 J. Chr15 is in *red* and the *Pvt1* locus is depicted in *blue line* with the 5′ and 3′ flanking FISH probes indicated. The inserted region of Chr12 is in *green* encompassing the region from Sα (junction 2) to Sμ (junction 1) as shown in Fig. 3d. The breakpoints of *Pvt1* locus are adjacent to each other in these two CTX junctions with only 143 bp deleted in the *Pvt1* locus

Interestingly, we identified a clonal *Igh* translocation in one G1XP lymphoma that did not involve *c-myc* locus since *c-myc* FISH probes had intact signals (Fig. 4c). Notably, this particular G1XP lymphoma (119 J) is aneuploid as evidenced by five copies of broken *Igh* locus and five copies of intact *c-myc* locus (Fig. 4c), suggesting the existence of novel translocation partners. Indeed, NGS data showed that this particular lymphoma harbored an *Igh*-*Pvt1* translocation (Fig. 3d). Next, we performed FISH analysis to validate the occurrence of this translocation. Surprisingly, we found that almost all metaphases harbored an intact *Pvt1* locus with 5′ and 3′ *Pvt1* probes always co-localized (Fig. 5a, left panel, and 5b), suggesting the absence of gross chromosomal structural alterations at the *Pvt1* locus. To reconcile the apparent discrepancy between NGS and FISH data, we used the 3′*Igh* and 3′*Pvt1* probes for FISH assay and found that these two probes co-localized in 100 % of metaphases analyzed (Fig. 5a, middle panel and 5b). In contrast, the 5′*Igh* and 5′*Pvt1* probes were not co-localized (Fig. 5a, right panel, and 5b). Thus, these data demonstrated that a piece of chromosome 12 was inserted into the *Pvt1* locus, which contained the centromeric portion of the *Igh* locus encompassing the region from Sα to Sμ (Fig. 5c). More importantly, these data implicate that G1XP lymphomas harbor complicated genomes including segmental translocations.

### G1XP lymphomas harbor ongoing DNA damage and a high level of clonal heterogeneity

**Fig. 6:**
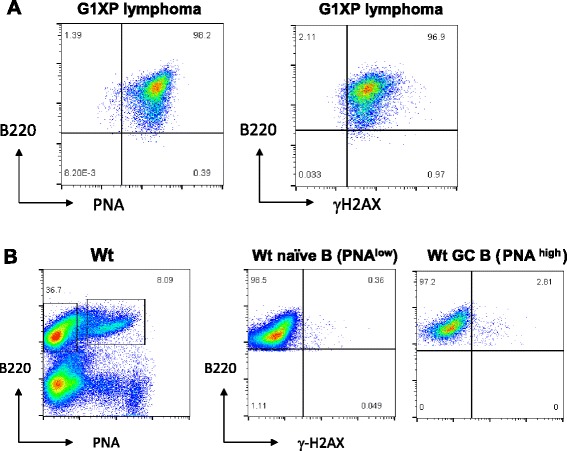
G1XP lymphomas harbor ongoing DNA damage. **a** FACS analysis of a GC B cell marker, PNA (*left panel*) and γ-H2AX foci staining (*right panel*) for G1XP lymphomas (*n* = 3 per group). **b** Wt splenocytes were isolated from immunized mice and stained for GC B cell markers, B220 and PNA. The level of γ-H2AX foci staining was shown for naïve B cells (B220^+^PNA^low^) and GC B cells (B220^+^PNA^high^). Representative FACS plots are shown from three independent experiments using independent tumor samples

We hypothesize that G1XP lymphomas probably harbor ongoing genomic instability that mediates the acquisition of chromosomal structural rearrangements. To test this hypothesis, we examined the DNA damage level in G1XP lymphomas via detecting the phosphorylation of H2AX (at Ser 139), a variant of H2A. H2AX phosphorylation occurs at sites flanking DSBs minutes after their induction, leading to the formation of distinct gamma-H2AX foci (γ-H2AX) [53]. Thus, γ-H2AX foci reflect the level of ongoing DSBs and are often used as an indicator of DSB formation. We used a flow cytometry-based method to measure γ-H2AX level in the G1XP lymphomas and in wt naïve (B220^+^PNA^low^) and GC (B220^+^PNA^high^) B cells as controls. Our data showed that G1XP lymphoma cells displayed a remarkably high level of γ-H2AX staining (Fig. 6a, right panel). In addition, this particular lymphoma expressed the GC B cell marker, PNA (Fig. 6a, left panel). In contrast, both the wt naïve and GC B cells exhibited a minimal level of γ-H2AX staining (Fig. 6b). Notably, the γ-H2AX level is consistently higher in GC B cells than naïve B cells (Fig. 6b). Thus, we conclude that G1XP lymphomas harbor ongoing DNA damage.

**Fig. 7:**
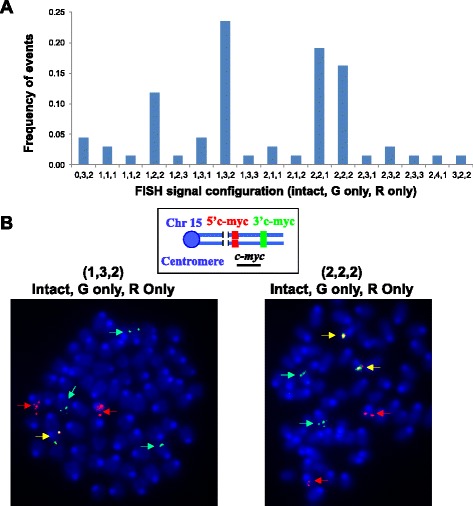
G1XP lymphomas harbor a high level of clonal heterogeneity. **a** Quantification of *c-myc* translocations in G1XP lymphoma samples (*n* = 3 independent tumor samples) analyzed by metaphase FISH. We subdivide the configuration of FISH signals into three categories: Intact: *green* and *red* probes co-localized; G only: *green* probe only (3′ *c-myc*), R only: *red* probe only (5′ *c-myc*). The frequency of different configurations of FISH signals was shown from one representative sample, and at least 150 metaphases were analyzed in total for each sample. **b** *Top*: Diagram of *c-myc* FISH probes. *Bottom*: Representative *c-myc* translocations showing different configurations of FISH signals. Numbers in parenthesis indicate the number of FISH signals for each category. Intact: *yellow arrows*; G only: *green arrows*; R only: *red arrows*

We predict that G1XP lymphomas may display a higher level of clonal heterogeneity. To test this possibility, we employed FISH assays to further analyze the lymphomas harboring the *c-myc* translocation in detail. Our analysis of these lymphomas showed that all metaphases indeed contained a *c-myc* translocation as shown by the split signals of 5′ and 3′ *c-myc* probes. Unexpectedly, we found that the configuration of FISH signals was highly heterogeneous since we observed an array of configurations that showed various numbers of intact, green (G) only or red (R) only FISH signals (Fig. 7a). It appeared that there were a few dominant subclones, for example, clone (1,3,2) and clone (2,2,2) (intact, G only, R only) (Fig. 7b), which occurred at a relatively high frequency, and many minor subclones carried various configurations of FISH signals (Fig. 7a). Thus, our G1XP lymphomas may provide a unique and novel model to study the mechanism of ongoing genomic instability and clonal heterogeneity.

## Discussion

A high level of genomic complexity and clonal heterogeneity may contribute to relapse or therapy resistance [54, 55]; however, key determinants regulating their generation have not been clearly addressed. In the current study, we establish a unique lymphoma model by specifically deleting *Xrcc4* and *Trp53* in the subset of B cells proposed to be prone to lymphomagenesis, namely, GC B cells [1]. Our mutant mouse B cells spontaneously develop B cell lymphomas, and we employed multiple approaches to characterize their genomic instability. Our studies reveal several important discoveries: (1) *Ig* loci translocations can be attributed to distinct mechanisms including AID- or RAG-associated DSBs in mature B cells; (2) AID-associated *Igh* translocations target oncogenes such as *c-myc* whereas RAG-associated translocations appear to involve random genomic loci; and (3) G1XP lymphomas harbor complicated genomes including segmental translocations, and exhibit a high level of ongoing DNA damage and clonal heterogeneity. Taken together, we propose that combined NHEJ and p53 defects may serve as an underlying mechanism for a high level of genomic complexity and clonal heterogeneity in cancers.

The NHEJ and p53 deficiency models have made significant contributions to our understanding of translocation and lymphomagenesis, more importantly, the molecular mechanism of DNA repair [15, 37, 39–43, 47]. Emerging evidence suggests that defects in DSB repair can lead to oncogenic genomic instability and, in support of this notion, mutations in DNA break repair factors are implicated in a number of human tumors, including breast, colon, and lung cancers [56]. In addition, somatic mutations in NHEJ factors have been identified in different types of human tumors including hypomorphic mutations of Artemis in EBV-associated lymphomas [57], mutations of Lig4 or XLF associated with non-Hodgkin’s diffuse large B cell lymphoma [58–60], and mutations of DNA-PKcs in glioblastoma and lung cancer [56]. *TP53* mutations were associated with human BL, its leukemic counterpart L3-type B cell acute lymphoblastic leukemia, B cell chronic lymphocytic leukemia (CLL), and, in particular, its stage of progression known as Richter’s transformation [61]. Richter syndrome (RS) is characterized by the transformation of CLL to high-grade non-Hodgkin’s lymphoma. Consistently, a recent study by performing a comprehensive molecular characterization of 86 pathologically proven RS reveals that *TP53* disruption (47.1 %) and *c-myc* abnormalities (26.2 %) were the most frequent alterations in RS [62], both of which are present in our models. Therefore, it is likely that defects in both NHEJ and p53 or in the modulators of these pathways may contribute to the development of human lymphomas, at least, a subset of them.

Our NGS data identified *Igh* translocation partners, *c-myc* and *Pvt-1*, which are often observed in BL and a subset of diffuse large B cell lymphomas [51, 63–67]. Thus, our model might provide a unique platform to better elucidate the molecular mechanisms of translocations in B cell lymphomagenesis. Prior studies demonstrate an important role of AID in promoting translocations in B cells [30]. We and others also prove that the NHEJ deficiency-induced *Igh* locus instability [15] or the generation of *Igh-c-myc* translocation is completely dependent on AID [68]. Consistently, we found that the majority of *Igh* translocations in G1XP lymphomas probably originated from AID-initiated DSBs, further solidifying its role in inducing *Igh* locus genomic instability. Furthermore, we find that half of *Ig* translocations occur in close proximity to V gene segments in the *Igh*, *Igκ*, or *Igλ* locus, strongly implicating these translocations catalyzed by RAGs. Notably, the partners of these *Ig* V gene translocations are random genetic loci or intergenic regions scattered all over the genome. We suggest that the generation of such translocations probably is largely influenced by mechanistic factors [69], such as the increased frequency of RAG-mediated DSBs at the *Igh* or *Igl* locus in the context of secondary V(D)J recombination. In this regard, these results are consistent with our previous findings that a small percentage of peripheral B cells harbor RAG-dependent *Igλ* breaks/translocations in the absence of *Xrcc4* [15]. Thus, our conclusion is further corroborated that mature B lymphocytes can undergo secondary V(D)J recombination, which may contribute to mature B cell lymphomagenesis.

Our data reveal that *Trp53* deficiency is essential to cause B cell lymphomas; however, *Trp53* deficiency per se does not increase the level of DSBs markedly. Thus, we propose that *Trp53* deficiency enhances the tolerance threshold of B cells for genomic instability induced by DNA repair deficiency in our model, thereby predisposing to lymphomagenesis. Consistent with our hypothesis, it has been shown that, in response to DSBs, p53 is phosphorylated and activated by ATM [70], then monitors DSBs in the context of G1 checkpoints, and signals arrest and/or apoptosis [71]. *Trp53* deficient mice usually succumb to thymic lymphomas that are aneuploid but lack translocations [72–75]. Of note, CD21cre-mediated deletion of *Trp53* in peripheral B cells results in the development of mature B cell lymphomas (IgM^+^) that lack recurrent clonal translocations involving *Ig* or *c-myc* loci [76]. Overall, these findings support our hypothesis that *Trp53* deficiency enables B cells to tolerate genomic instability. Furthermore, we propose that the regulation of genomic instability tolerance is more p53-dependent in B cells than in other cell lineages. This notion is supported by the findings that NHEJ/p53 germline deficient mice developed only pro-B cell lymphomas [39, 43]. Thus, our unique mouse model may facilitate the discovery of critical components of p53-mediated effector cascades that regulate genomic instability tolerance. Furthermore, we were able to establish cell lines from our lymphoma model (data not shown), which would facilitate subsequent studies. Addressing these fundamental questions potentially identifies targets that specifically attack cancer cells with unstable genomes, while leaving genetically stable normal cells unaffected.

With regard to the potential of our model in clinical applications, such as biomarkers for diagnosis and therapy [77], we suggest that our unique model might potentially provide novel insights into the biomarker development in predicting the onset of the B cell lymphomas, given that this lymphoma model has a relatively long latency and low penetrance. In addition, novel therapies have been developed rapidly to treat B cell lymphomas or CLL, for example, Ibrutinib and new agents are effective for *TP53* mutant lymphoma cells; thus, there is the potential of clinical applications of our lymphoma model for testing new agents [78–80]. Mechanistically, it would be of interest to elucidate which signaling pathway is required for the survival of these lymphoma cells.

## Conclusions

Deletion of *Xrcc4* and *Trp53* via Cγ1Cre leads to novel B cell lymphomas that appear to derive from GC B cells. These B cell lymphomas harbor ongoing DNA damage and exhibit a high level of clonal heterogeneity for characteristic *c-myc* translocations. We propose that combined NHEJ and p53 defects may serve as an underlying mechanism for a high level of genomic complexity and clonal heterogeneity in cancers.

## Methods

### Generation of mouse models

Cγ1Cre knock-in (KI) mice [49], *Xrcc4* [37], or *Trp53* [81] conditional knock-out (KO) mice were generated previously. These mice were in mixed genetic background of C57BL/6, 129/Ola, and FVB/N [37, 49, 81]. Animal work was approved by the Institutional Animal Care and Use Committee of University of Colorado Anschutz Medical Campus (Aurora, CO), National Jewish Health (Denver, CO), and Children’s Hospital in Boston (Boston, MA).

### B cell culture, FISH, and Southern Blot analysis

Splenic B cells were isolated from naïve mice, purified by negative selection kit (Stem Cell Technologies, Canada), activated with anti-CD40 and IL4 as described previously [82], and collected 4 days after culture for metaphase preparation and FISH analysis. FISH analysis was performed with specific BAC probes as previously described [82] (see details in Additional file 1). Genomic DNA was isolated from tumor masses or normal tissues from control mice, and Southern blotting was performed as previously described [41].

### H&E staining, flow cytometry, and H2AX foci staining

Tumor masses usually presented in the abdomen and were adjacent to gut-associated lymphoid tissues such as mesenteric lymph nodes or Peyer’s patch. Tumors were dissected, fixed in 10 % formalin, and processed for H&E histology staining. Tumor single-cell suspensions were prepared and subjected to flow cytometry for phenotypic characterization. Samples were analyzed using a FACSCalibur (BD Bioscience), and FACS analysis was performed with FlowJo software. Single-cell suspensions prepared from tumors or wt naïve or GC B cells were stained for γ-H2AX foci according to the manufacturer’s instructions (BD Bioscience). The generation of splenic GC B cells was induced by in vivo immunization as described previously [82].

### NGS library preparation, sequencing platform, and data analysis

Tumor DNA samples were employed to generate the NGS paired-end library using the standard TruSeq DNA library preparation kit (Illumina, San Diego, CA). The libraries were subjected to whole genome sequencing on the Illumina Hi-Seq 2000 platform (pair-ended, 2 × 100 bp per read). Details of NGS analysis are provided in the Additional file 1 including usage of CREST software [83] and generation of Circos plots [84].

## Additional files

Additional file 1:
**Figure S1. Establishment of G1XP lymphoma model**. (A) Specific Xrcc4 deletion via Cγ1Cre in GC B cells. PCR analysis of genomic DNA isolated from bone marrow (BM), thymus, sorted naïve B cells (B220+PNAlow), and GC B cells (B220+PNAhigh) of immunized Cγ1Cre KI or Cγ1CreXc/+ mice. The middle band is non-specific amplification of PCR reaction for BM, thymus and B220+PNAlow B cells. GC B cells (B220+PNAhigh) show the complete deletion of Xrcc4 floxed allele. Splenocytes were stained using anti-B220 and anti-PNA antibodies. (B) Kaplan Meier survival curve of the first cohort of mice including Cγ1CreXcPc/+mice (n=19, blue line), Cγ1CreXc/+Pc mice (n=20, green line) and Cγ1CreXcPc mice (n=47, red line). (C) Southern Blot analysis of 1st cohort of Cγ1CreXcPc mice. G1XP lymphomas harbor clonal JH rearrangements. Southern blot analyses for JH rearrangements in G1XP lymphoma genomic DNA employing EcoRI digestion and the JH4 probe. Germline (GL) bands are indicated (arrow heads). Asterisks (*) indicate rearranged JH locus in lymphoma DNA. MLN stands for mesenteric lymph node. **Figure S2**. G1XP lymphomas harbor somatic hypermutations. Germline sequences from either the JH1-4 or JH3-4 region were aligned with G1XP lymphoma sequences. The mismatched sequences (in red font) at the beginning of the sequences are derived from rearranged VD or D regions. To exclude the effects of V(D)J recombination in sequence diversity, we only identified the mutations in JH exon or intron regions that could be unequivocally aligned to the germline sequences. The rearranged JH exon regions are underlined for each individual G1XP lymphoma samples, and the mutations are marked with “*”. **Figure S3**. G1XP lymphomas harbor normal or aberrant V(D)J recombination junctions. NGS data showed the presence of normal D-J (junction 1) and V-D-J (junction 2) junctions at the Igh locus or aberrant V-J junction (junction 3) at Igλ locus.V, D, and J exons were labeled and depicted as orange bars.MH: micro-homology. **Figure S4**.Clonal Igλtranslocations in G1XP lymphoma. Top: schematics of Igλ locus on chromosome 16. Bottom: Metaphases from G1XP lymphoma samples were analyzed by FISH for hybridization to Igλ probes as indicated in the schematics. Representative Igλ translocations in G1XP lymphomas are shown. **Table S1**. Phenotypic characterization of G1XP lymphoma via flow cytometry. **Table S2**. Summary of translocation involved Igh and Igl genes. **Table S3**.Validation of Translocations by PCR, Sanger Sequencing or FISH.(PDF 1221 kb)
