# Supplementary material for: Combined deletion of Xrcc4 and Trp53 in mouse germinal center B cells leads to novel B cell lymphomas with clonal heterogeneity
Source: J Hematol Oncol. 2016 Jan 7;9:2. doi: 10.1186/s13045-015-0230-5 (PMC4704435; doi:10.1186/s13045-015-0230-5)
Supplement: Additional file 1: — Figure S1. Establishment of G1XP lymphoma model. (A) Specific Xrcc4 deletion via Cγ1Cre in GC B cells. PCR analysis of genomic DNA isolated from bone marrow (BM), thymus, sorted naïve B cells (B220+PNAlow), and GC B cells (B220+PNAhigh) of immunized Cγ1Cre KI or Cγ1CreXc/+ mice. The middle band is non-specific amplification of PCR reaction for BM, thymus and B220+PNAlow B cells. GC B cells (B220+PNAhigh) show the complete deletion of Xrcc4 floxed allele. Splenocytes were stained using anti-B220 and anti-PNA antibodies. (B) Kaplan Meier survival curve of the first cohort of mice including Cγ1CreXcPc/+mice (n=19, blue line), Cγ1CreXc/+Pc mice (n=20, green line) and Cγ1CreXcPc mice (n=47, red line). (C) Southern Blot analysis of 1st cohort of Cγ1CreXcPc mice. G1XP lymphomas harbor clonal JH rearrangements. Southern blot analyses for JH rearrangements in G1XP lymphoma genomic DNA employing EcoRI digestion and the JH4 probe. Germline (GL) bands are indicated (arrow heads). Asterisks (*) indicate rearranged JH locus in lymphoma DNA. MLN stands for mesenteric lymph node. Figure S2. G1XP lymphomas harbor somatic hypermutations. Germline sequences from either the JH1-4 or JH3-4 region were aligned with G1XP lymphoma sequences. The mismatched sequences (in red font) at the beginning of the sequences are derived from rearranged VD or D regions. To exclude the effects of V(D)J recombination in sequence diversity, we only identified the mutations in JH exon or intron regions that could be unequivocally aligned to the germline sequences. The rearranged JH exon regions are underlined for each individual G1XP lymphoma samples, and the mutations are marked with “*”. Figure S3. G1XP lymphomas harbor normal or aberrant V(D)J recombination junctions. NGS data showed the presence of normal D-J (junction 1) and V-D-J (junction 2) junctions at the Igh locus or aberrant V-J junction (junction 3) at Igλ locus.V, D, and J exons were labeled and depicted as orange bars.MH: micro-homology. Fig [file 13045_2015_230_MOESM1_ESM.pdf]

SUPPLEMENTAL FIGURE LEGENDS

Figure S1. Establishment of G1XP lymphoma model. (A) Specific *Xrcc4* deletion via Cγ1Cre in GC B cells. PCR analysis of genomic DNA isolated from bone marrow (BM), thymus, sorted naïve B cells ($B220^{+}PNA^{low}$), and GC B cells ($B220^{+}PNA^{high}$) of immunized Cγ1Cre KI or Cγ1Cre$X^{c/+}$ mice. The middle band is non-specific amplification of PCR reaction for BM, thymus and $B220^{+}PNA^{low}$ B cells. GC B cells ($B220^{+}PNA^{high}$) show the complete deletion of *Xrcc4* floxed allele. Splenocytes were stained using anti-B220 and anti-PNA antibodies. **(B)** Kaplan Meier survival curve of the first cohort of mice including $C\gamma 1CreX^{c}P^{c/+}$ mice (n=19, blue line), $C\gamma 1CreX^{c/+}P^{c}$ mice (n=20, green line) and $C\gamma 1CreX^{c}P^{c}$ mice (n=47, red line). **(C)** Southern Blot analysis of 1st cohort of $C\gamma 1CreX^{c}P^{c}$ mice. G1XP lymphomas harbor clonal J_H rearrangements. Southern blot analyses for J_H rearrangements in G1XP lymphoma genomic DNA employing EcoRI digestion and the J_{H4} probe. Germline (GL) bands are indicated (arrow heads). Asterisks (∗) indicate rearranged J_H locus in lymphoma DNA. MLN stands for mesenteric lymph node.

Figure S2. G1XP lymphomas harbor somatic hypermutations. Germline sequences from either the J_{H1-4} or J_{H3-4} region were aligned with G1XP lymphoma sequences. The mismatched sequences (in red font) at the beginning of the sequences are derived from rearranged VD or D regions. To exclude the effects of V(D)J recombination in sequence diversity, we only identified the mutations in J_H exon or intron regions that could be unequivocally aligned to the germline sequences. The rearranged J_H exon regions are

underlined for each individual G1XP lymphoma samples, and the mutations are marked with "*".

Figure S3. G1XP lymphomas harbor normal or aberrant V(D)J recombination junctions. NGS data showed the presence of normal D-J (junction 1) and V-D-J (junction 2) junctions at the *Igh* locus or aberrant V-J junction (junction 3) at *Igλ* locus. V, D, and J exons were labeled and depicted as orange bars. MH: micro-homology.

Figure S4. Clonal *Igλ* translocations in G1XP lymphoma. Top: schematics of *Igλ* locus on chromosome 16. **Bottom:** Metaphases from G1XP lymphoma samples were analyzed by FISH for hybridization to *Igλ* probes as indicated in the schematics. Representative *Igλ* translocations in G1XP lymphomas are shown.

SUPPLEMENTAL METHODS

Generation of mouse models.

Cγ1Cre knock-in (KI) mice have the IRES-Cre cDNA inserted into the 3' UTR region of Cγ1 exon without affecting IgG1 expression [1]. We intercrossed these strains to generate the *Cγ1Cre/Xrcc4*$^{c/c\ or\ c/-}$*/p53*$^{c/c}$ cohort and control mice for survival study, and *Cγ1Cre/Xrcc4*$^{c/c\ or\ c/-}$ or *Cγ1Cre/p53*$^{c/c}$ single deficient mice for *Igh* locus instability analysis. The genetic background of these mice is mixed with 129, B6, and FVB/N [1-3].

FACS

Antibodies used for flow cytometry were obtained from BD Bioscience including, anti-B220-PE, anti-B220-FITC, anti-B220-APC, anti-CD23, anti-CD22, anti-CD24, anti-CD38, anti-CD43-PE, anti-CD93-PE, anti-CD138, anti-FAS, anti-IgM-FITC, anti-IgG1-FITC, anti-IgG2a/2b-FITC, anti-IgA-PE, anti-IgD, and anti-Igκ. Also used were anti-Igλ (Southern Biotechnology) and anti-PNA-FITC (Vector).

Probes for southern blotting and BAC probes for FISH

J_{H4} probe was the HindIII-EcoRI (1.6kb) fragment of 3' J_{H4} region. FISH probes were as follows: a BAC that covered the 3' region of the *Igh* locus encompassing *3' Igh* enhancer and 100kb downstream (3' *Igh* BAC), a BAC just upstream of the *Igh* V_H region (5' *Igh* BAC) as described previously [4]. All BACs outlined below were obtained from the BACPAC CHORI database. BACs for *Igλ* regions are RP23-382P9 (5'*Igλ*) and RP23-374P12 (3'*Igλ*). BACs for *c-myc* regions are RP24-434C10 (5' *c-myc*) and RP23-457I7

(3' *c-myc*). BACs for *Pvt-1* regions are RP23-98D8 (5' *Pvt-1*) and RP24-413E20 (3' *Pvt-1*). In all FISH experiments, intact loci show co-localization of the red and green probes, while split red and green signals are scored as broken loci. Broken loci can be free, with the centromeric and telomeric portion of the locus either present in the metaphases or lost. Alternatively, broken loci (both the centromeric and telomeric parts or only one of them) can be involved in translocations with other chromosomes.

Ig cloning and sequencing

VDJ exon sequencing and cloning were performed as described previously [5]. For mutational analysis, Iproof high fidelity polymerase was employed (Biorad). Sequences were analyzed with Lasergene DNA-STAR/SeqMan software and were aligned with the corresponding genomic sequences of J_H regions (accession number: AJ851868, http://www.ncbi.nlm.nih.gov/nuccore/126349412).

NGS analysis including CREST software and generation of Circos plots.

We have another study to focus on the analysis of NGS data (Chen et al., manuscript in preparation) in which we sequenced wt control B cells from various genetic backgrounds to control for NGS pipelines. In the current study, we sequenced 6 tumor samples, 46J, 90J, 119J, 125J, 196J, and 202J, with coverages ranging between 30× and 40× in depth. The raw sequencing data was analyzed via CREST software [6] and aligned to mouse mm9 reference sequences. CREST software has been employed to analyze NGS data of human leukemia or lymphoma samples [6]. In addition, CREST has been applied to analyze NGS data obtained from other types of human cancers using the

same Illumina HiSeq 2000 platform as we did, which indeed detected structural variations [7, 8]. Therefore, we used this software to identify structural variations in our mouse lymphoma samples. The list of potential rearrangements provided by CREST was extensively filtered for those most likely to be true novel rearrangements. Each variant was required to be unique to a single tumor sample, and to have evidence of soft-clipping reads at each contributing breakpoint end. In addition, majority of the CTXs was supported by pair-end sequencing analysis. Lastly, we were able to confirm the occurrence of NGS-identified CTXs with independent methodology (e.g. FISH or PCR). Circos plots were generated using the software described previously [9]. The Integrative Genome Viewer (IGV) software was employed [10] to visually examine the location of CTX events in the context of annotated genetic loci.

Reference

1. Casola S, Cattoretti G, Uyttersprot N, Koralov SB, Seagal J, Hao Z, Waisman A, Egert A, Ghitza D, Rajewsky K: **Tracking germinal center B cells expressing germ-line immunoglobulin gamma1 transcripts by conditional gene targeting**. *Proc Natl Acad Sci U S A* 2006, **103**(19):7396-7401.
2. Jonkers J, Meuwissen R, van der Gulden H, Peterse H, van der Valk M, Berns A: **Synergistic tumor suppressor activity of BRCA2 and p53 in a conditional mouse model for breast cancer**. *Nat Genet* 2001, **29**(4):418-425.
3. Yan CT, Boboila C, Souza EK, Franco S, Hickernell TR, Murphy M, Gumaste S, Geyer M, Zarrin AA, Manis JP, Rajewsky K, Alt FW: **IgH class switching and translocations use a robust non-classical end-joining pathway**. *Nature* 2007, **449**(7161):478-482.
4. Franco S, Gostissa M, Zha S, Lombard DB, Murphy MM, Zarrin AA, Yan C, Tepsuporn S, Morales JC, Adams MM, Lou Z, Bassing CH, Manis JP, Chen J, Carpenter PB, Alt FW: **H2AX prevents DNA breaks from progressing to chromosome breaks and translocations**. *Mol Cell* 2006, **21**(2):201-214.
5. Wang JH, Alt FW, Gostissa M, Datta A, Murphy M, Alimzhanov MB, Coakley KM, Rajewsky K, Manis JP, Yan CT: **Oncogenic transformation in the absence of Xrcc4 targets peripheral B cells that have undergone editing and switching**. *J Exp Med* 2008, **205**(13):3079-3090.
6. Wang J, Mullighan CG, Easton J, Roberts S, Heatley SL, Ma J, Rusch MC, Chen K, Harris CC, Ding L, Holmfeldt L, Payne-Turner D, Fan X, Wei L, Zhao D, Obenauer JC, Naeve C, Mardis ER, Wilson RK, Downing JR, Zhang J: **CREST maps somatic structural variation in cancer genomes with base-pair resolution**. *Nat Methods* 2011, **8**(8):652-654.
7. Wang Y, Waters J, Leung ML, Unruh A, Roh W, Shi X, Chen K, Scheet P, Vattathil S, Liang H, Multani A, Zhang H, Zhao R, Michor F, Meric-Bernstam F, Navin NE: **Clonal evolution in breast cancer revealed by single nucleus genome sequencing**. *Nature* 2014, **512**(7513):155-160.
8. Xie T, Cho YB, Wang K, Huang D, Hong HK, Choi YL, Ko YH, Nam DH, Jin J, Yang H, Fernandez J, Deng S, Rejto PA, Lee WY, Mao M: **Patterns of somatic alterations between matched primary and metastatic colorectal tumors characterized by whole-genome sequencing**. *Genomics* 2014, **104**(4):234-241.
9. Krzywinski M, Schein J, Birol I, Connors J, Gascoyne R, Horsman D, Jones SJ, Marra MA: **Circos: an information aesthetic for comparative genomics**. *Genome Res* 2009, **19**(9):1639-1645.
10. Thorvaldsdottir H, Robinson JT, Mesirov JP: **Integrative Genomics Viewer (IGV): high-performance genomics data visualization and exploration**. *Briefings in bioinformatics* 2013, **14**(2):178-192.

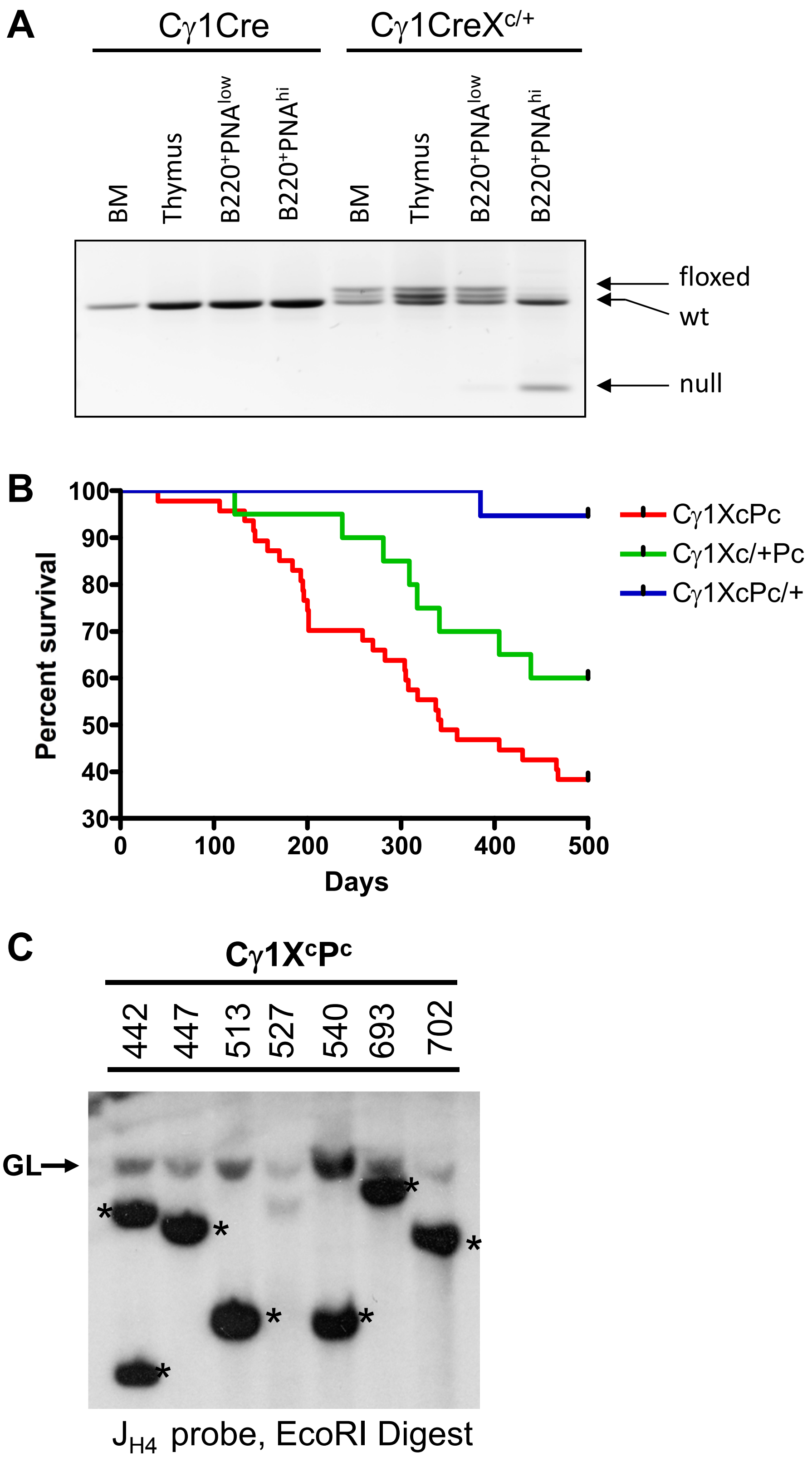
A
Cγ1Cre
Cγ1CreX c/+
BM
Thymus
B220+PNAlow
B220+PNAhi
BM
Thymus
B220+PNAlow
B220+PNAhi
floxed
wt
null
B
Percent survival
Days
Cγ1XcPc
Cγ1Xc/+Pc
Cγ1XcPc/+
C
Cγ1XcPc
442
447
513
527
540
693
702
GL
JH4 probe, EcoRI Digest

Figure S1A-C

G1XP 540: 13 mutations in J_{H3} intronic region (marked by "*")

```
                                       10        20        30        40        50        60        70        80
                              atttattgtcaggggtctaatcAYTRTKGTYACRAcKGKGCCTGGTTTGCTTACTGGGGCCAAGGGACTCTGGTCACTGTCTCTG
JH3-4 region(1>1885)       →  atttattgtcaggggtctaatcattgttgtcacaa-tgtgcctggtttgcttactggggccaagggactctggtcactgtctctg
540V7183-JH4.ab1(10>745)   ←                        ACTATGGTTACGACGGGGCCTGGTTTGCTTACTGGGGCCAAGGGACTCTGGTCACTGTCTCTG
                                                                                JH3 exon

                                 90       100       110       120       130       140       150       160       170
                              CAGGTGAGTCCTAAYTTCTCCCATTSTARATGCATSTTGGGGGGATTCTGAGCCTTCAGGAYCAMGATTCKCTGCAAAYGGGAAT
JH3-4 region(1>1885)       →  caggtgagtcctaacttctcccattctaaatgcatgttggggggattctgagccttcaggaccaagattctctgcaaacgggaat
540V7183-JH4.ab1(10>745)   ←  CAGGTGAGTCCTAATTTCTCCCATTGTAGATGCATCTTGGGGGGATTCTGAGCCTTCAGGATCACGATTCGCTGCAAATGGGAAT
                                            *          *  *      *                         *  *     *       *
                              JH3 exon
                                      180       190       200       210       220       230       240       250
                              SAAGATTCAACCCCTTTGTCCCAAAGTTGAGACATGGGTCTGGGTCAGGGACTCTCTGCCTGCTGGTCTGTGGTGACATTWGAAC
JH3-4 region(1>1885)       →  caagattcaacccctttgtcccaaagttgagacatgggtctgggtcagggactctctgcctgctggtctgtggtgacattagaac
540V7183-JH4.ab1(10>745)   ←  GAAGATTCAACCCCTTTGTCCCAAAGTTGAGACATGGGTCTGGGTCAGGGACTCTCTGCCTGCTGGTCTGTGGTGACATTTGAAC
                              *                                                                               *
                                260       270       280       290       300       310       320       330       340
                              TGAAGTATGATGAAGGATCTGCCAGAACTGAARCTTGAAGTCTGAGGCAGAATCTTGTCCAGGGTCTATCGGACTCTTGTGAGAA
JH3-4 region(1>1885)       →  tgaagtatgatgaaggatctgccagaactgaagcttgaagtctgaggcagaatcttgtccagggtctatcggactcttgtgagaa
540V7183-JH4.ab1(10>745)   ←  TGAAGTATGATGAAGGATCTGCCAGAACTGAAACTTGAAGTCTGAGGCAGAATCTTGTCCAGGGTCTATCGGACTCTTGTGAGAA
                                                              *
                                      350       360       370       380       390       400       410       420
                              TTAGGGGCTGACAGTTGATGGTGACAATTTCAGGGTCAGTGACTGTCTGGTTTCTCTGAGGTGAGGCTGGAATATAGGTCACCTT
JH3-4 region(1>1885)       →  ttaggggctgacagttgatggtgacaatttcagggtcagtgactgtctggtttctctgaggtgaggctggaatataggtcacctt
540V7183-JH4.ab1(10>745)   ←  TTAGGGGCTGACAGTTGATGGTGACAATTTCAGGGTCAGTGACTGTCTGGTTTCTCTGAGGTGAGGCTGGAATATAGGTCACCTT

                                430       440       450       460       470       480       490       500       510
                              GAAGACTTAAGAGGGGTCCAGGGGGCTTCTGCACAGGCAGGGAACAGAATGTGGAACAATGACTTGAATGGTTGATTCTTGTGTG
JH3-4 region(1>1885)       →  gaagacttaagaggggtccagggggcttctgcacaggcagggaacagaatgtggaacaatgacttgaatggttgattcttgtgtg
540V7183-JH4.ab1(10>745)   ←  GAAGACTTAAGAGGGGTCCAGGGGGCTTCTGCACAGGCAGGGAACAGAATGTGGAACAATGACTTGAATGGTTGATTCTTGTGTG

                                      520       530       540       550       560       570       580       590
                              ACACCAGGAATTGGCATAATGTCTGAGTTGCCCAGGGGTGATTCTAGTCAGACTCTGGGGTTTTTGTCGGRTATAGAGGAAAAAT
JH3-4 region(1>1885)       →  acaccaggaattggcataatgtctgagttgcccaggggtgattctagtcagactctggggtttttgtcgggtatagaggaaaaat
540V7183-JH4.ab1(10>745)   ←  ACACCAGGAATTGGCATAATGTCTGAGTTGCCCAGGGGTGATTCTAGTCAGACTCTGGGGTTTTTGTCGGATATAGAGGAAAAAT
                                                                                      *
                                600       610       620       630       640       650       660       670       680
                              CCACTATTGTGATTACTATGCTATGGACTACTGGGGTCAAGGAACCTCAGTCACCGTCTCCTCAGGTMAGAATGGCCTCTCCAGG
JH3-4 region(1>1885)       →  ccactattgtgattactatgctatggactactggggtcaaggaacctcagtcaccgtctcctcaggtaagaatggcctctccagg
540V7183-JH4.ab1(10>745)   ←  CCACTATTGTGATTACTATGCTATGGACTACTGGGGTCAAGGAACCTCAGTCACCGTCTCCTCAGGTCAGAATGGCCTCTCCAGG
                                                                                 *
```

Figure S2

G1XP 447: 3 mutations in J_{H2} exon and intron region (marked by "*")

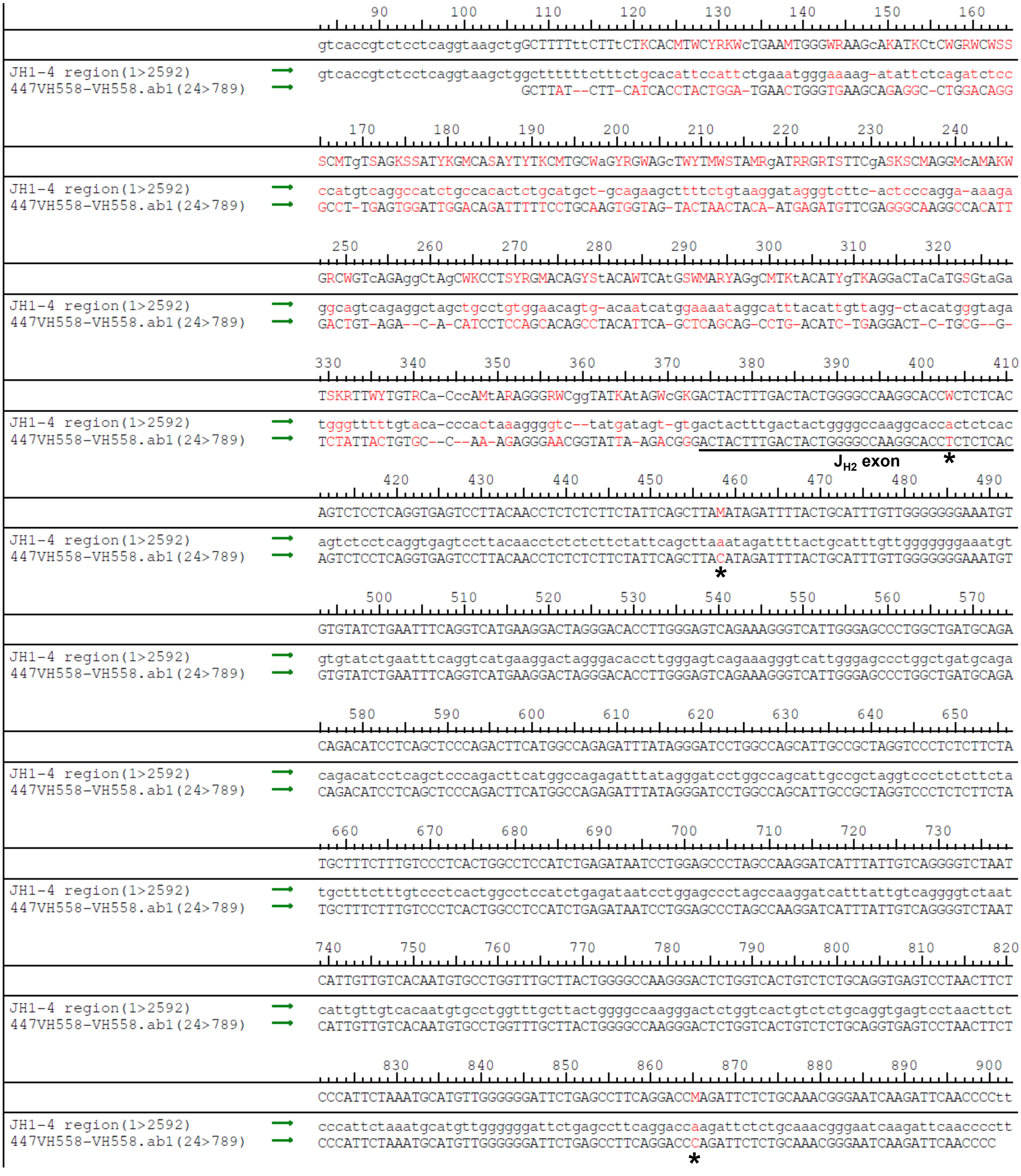

Figure S2, cont

G1XP 693: 1 mutation in J_{H1} intronic region (marked by "*")

```
                                 10        20        30        40        50        60        70        80
                             YGGMSAKaGTWTTASTRTaGSAASASARGcaGRACWGRGACtGWGctAcTGGTACTTCGATGTCTGGGGCGCAGGGACCACG
693V7183-V7183.ab1(20>531)   CGGCCAT-GTATTACTGT-GCAAGACAAG--GGACTGGGAC-GAG--A-TGGTACTTCGATGTCTGGGGCGCAGGGACCACG
JH1-4 region(1>2592)         tggagagagtttagtataggaacagaggcagaacagagactgtgctactggtacttcgatgtctggggcgcagggaccacg
                                                                             JH1 exon
                                 90       100       110       120       130       140       150       160
                             GTCACCGTCTCCTCAGGTAAGCTGGCTTTTTTCTTTCTGCACATTCCATTCTGAAATGGGAAAAGATATTCTCAGATCTCCC
693V7183-V7183.ab1(20>531)   GTCACCGTCTCCTCAGGTAAGCTGGCTTTTTTCTTTCTGCACATTCCATTCTGAAATGGGAAAAGATATTCTCAGATCTCCC
JH1-4 region(1>2592)         gtcaccgtctcctcaggtaagctggcttttttctttctgcacattccattctgaaatgggaaaagatattctcagatctccc

                                170       180       190       200       210       220       230       240
                             CATGTCAGGCCATCTGCCACACTCTGCATGCTGCAGAAGCTTTTCTGTAARGATAGGGTCTTCACTCCCAGGAAAAGAGGCA
693V7183-V7183.ab1(20>531)   CATGTCAGGCCATCTGCCACACTCTGCATGCTGCAGAAGCTTTTCTGTAAAGATAGGGTCTTCACTCCCAGGAAAAGAGGCA
JH1-4 region(1>2592)         catgtcaggccatctgccacactctgcatgctgcagaagcttttctgtaaggatagggtcttcactcccaggaaaagaggca
                                                                              *
                               250       260       270       280       290       300       310       320
                             GTCAGAGGCTAGCTGCCTGTGGAACAGTGACAATCATGGAAAATAGGCATTTACATTGTTAGGCTACATGGGTAGATGGGTT
693V7183-V7183.ab1(20>531)   GTCAGAGGCTAGCTGCCTGTGGAACAGTGACAATCATGGAAAATAGGCATTTACATTGTTAGGCTACATGGGTAGATGGGTT
JH1-4 region(1>2592)         gtcagaggctagctgcctgtggaacagtgacaatcatggaaaataggcatttacattgttaggctacatgggtagatgggtt

                             330       340       350       360       370       380       390       400       410
                             TTTGTACACCCACTAAAGGGGTCTATGATAGTGTGACTACTTTGACTACTGGGGCCAAGGCACCACTCTCACAGTCTCCTCA
693V7183-V7183.ab1(20>531)   TTTGTACACCCACTAAAGGGGTCTATGATAGTGTGACTACTTTGACTACTGGGGCCAAGGCACCACTCTCACAGTCTCCTCA
JH1-4 region(1>2592)         tttgtacacccactaaaggggtctatgatagtgtgactactttgactactggggccaaggcaccactctcacagtctcctca

                                  420       430       440       450       460       470       480       490
                             GGTGAGTCCTTACAACCTCTCTCTTCTATTCAGCTTAAATAGATTTTACTGCATTTGTTGGGGGGAAATGTGTGTATCTGA
693V7183-V7183.ab1(20>531)   GGTGAGTCCTTACAACCTCTCTCTTCTATTCAGCTTAAATAGATTTTACTGCATTTGTTGGGGGGAAATGTGTGTATCTGA
JH1-4 region(1>2592)         ggtgagtccttacaacctctctcttctattcagcttaaatagattttactgcatttgttggggggaaatgtgtgtatctga
```

Figure S2, cont

Junction 1: 46J_DEL_chr12:114667724 to chr12:114720381

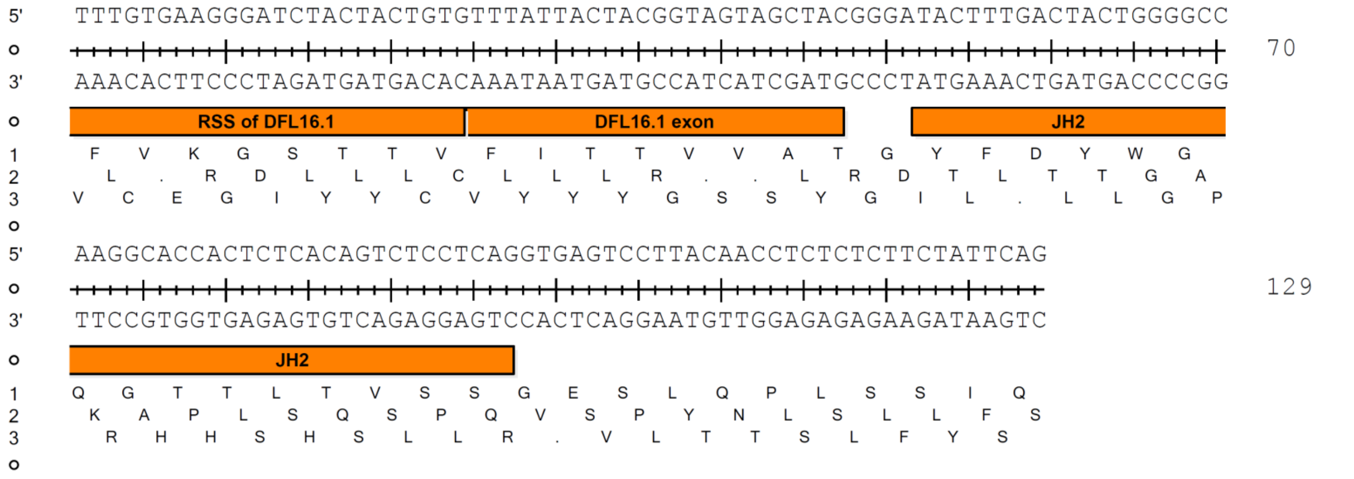

Junction 2: 125J_DEL_chr12:114666778 to chr12: 114816716

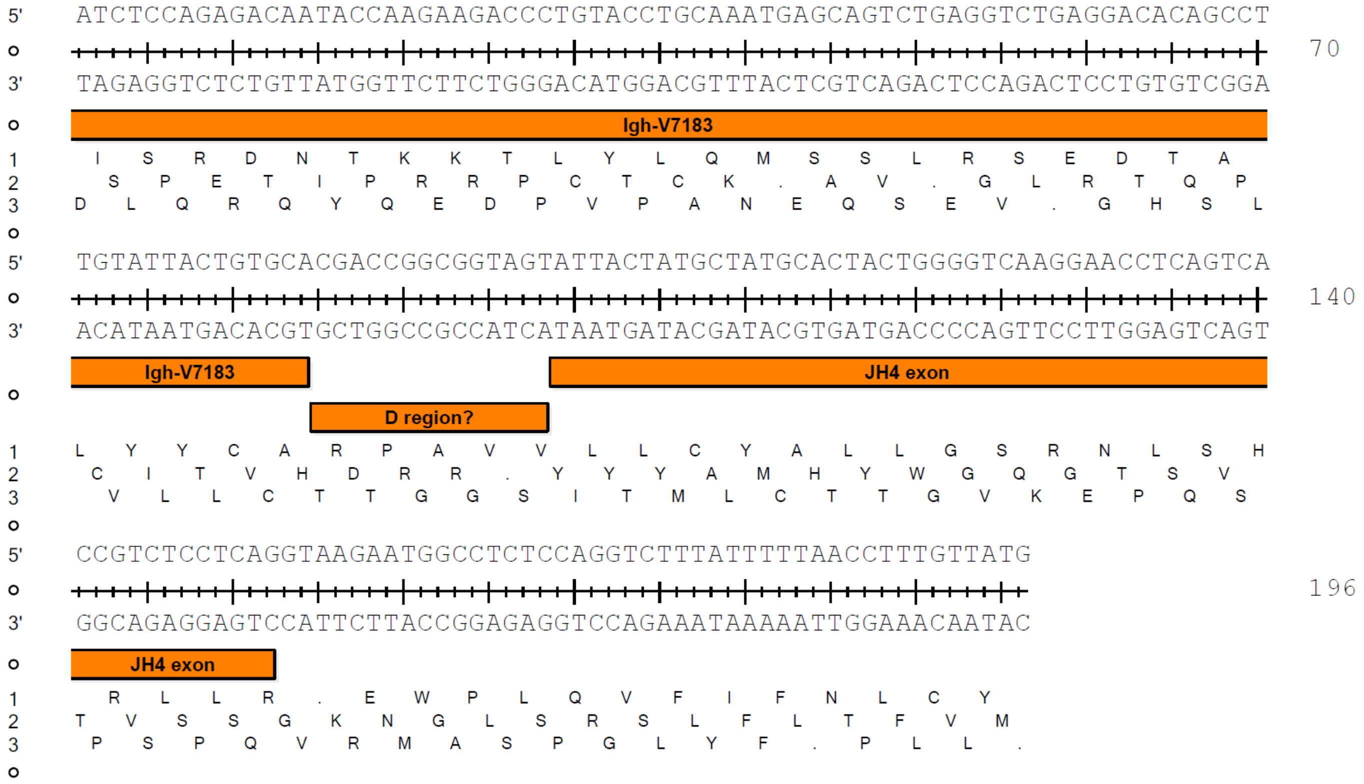

Figure S3

Junction 3: 202J_DEL_chr16: 19067135 to chr16:19085148

```
5' CAACAACCGAGCTCCAGGTGTTCCTGCCAGATTCTCAGGCTCCCTGATTGGAGACAAGGCTGCCCTCACCATCACAGGGG  80
3' GTTGTTGGCTCGAGGTCCACAAGGACGGTCTAAGAGTCCGAGGGACTAACCTCTGTTCCGACGGGAGTGGTAGTGTCCCC
   IgL-VL1-truncated exon
1  Q Q P S S R C S C Q I L R L P D W R Q G C P H H H R G
2  N N R A P G V P A R F S G S L I G D K A A L T I T G
3  S T T E L Q V F L P D S Q A P . L E T R L P S P S Q G

5' CACAGACTGAGGATGAGGTAAGTGGCTTTAATGCTTCTTCCTAATAAGTCCAGGCCTTGTTATCTTGCAAGGGTCATTTA  160
3' GTGTCTGACTCCTACTCCATTCACCGAAATTACGAAGAAGGATTATTCAGGTCCGGAACAATAGAACGTTCCCAGTAAAT
   IgL-VL1-truncated exon   JL3 intron
   MH
1  T D . G . G K W L . C F F L I S P G L V I L Q G S F
2  A Q T E D E V S G F N A S S . . V Q A L L S C K G H L
3  H R L R M R . V A L M L L P N K S R P C Y L A R V I Y

5' TCTCTCTGGAAAGCTTTTCTCATCTGCT  188
3' AGAGAGACCTTTCGAAAAGAGTAGACGA
   JL3 intron
1  I S L E S F S H L L
2  S L W K A F L I C
3  L S G K L F S S A
```

Figure S3, cont

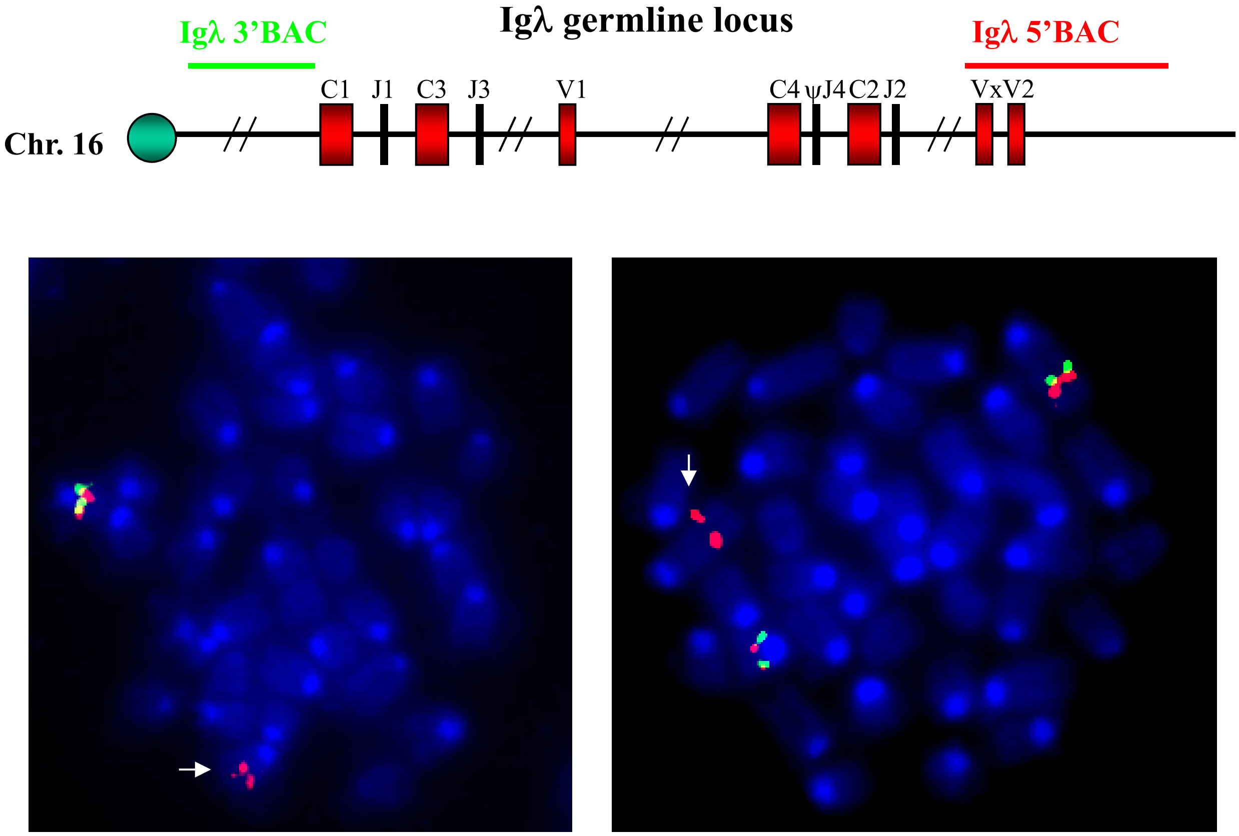

Igλ 3'BAC
Igλ germline locus
Igλ 5'BAC
C1
J1
C3
J3
V1
C4
ψJ4
C2
J2
Vx
V2
Chr. 16

Figure S4

Table S1: Phenotypic characterization of G1XP lymphoma via flow cytometry

ID	B220	CD22	CD23	CD24	CD38	CD43	CD93	CD138	Igκ	Igλ	IgM	IgA	IgG1	IgG2a/2b	FAS	PNA
**46J**	positive	negative	negative	very high	positive	positive	very high	positive	negative	negative	negative	negative	negative	negative	negative	negative
**119J**	positive	negative	intermediate	positive	positive	positive	positive	positive	negative	positive	negative	ND	positive	ND	negative	ND
**125J**	positive	pos and neg	negative	positive	positive	positive	positive	positive	negative	negative	negative	negative	negative	negative	negative	negative
**196J**	positive	negative	negative	positive	positive	positive	positive	positive	negative	positive	negative	negative	positive	negative	negative	positive
**202J**	positive	negative	negative	positive	positive	positive	positive	positive	negative	positive	negative	negative	positive	negative	negative	positive
**105J**	positive	negative	negative	positive	positive	positive	positive	positive	positive	negative	positive	negative	negative	negative	negative	negative
**224J**	positive	negative	negative	positive	positive	positive	positive	positive	negative	negative	negative	negative	negative	negative	negative	positive
**1J**	positive	negative	negative	positive	positive	positive	positive	positive	negative	negative	negative	negative	negative	negative	negative	positive
**164J**	positive	positive	negative	positive	positive	positive	positive	positive	intermediate	negative	negative	negative	negative	intermediate	pos and neg	positive
**76J**	positive	positive	negative	positive	positive	positive	positive	positive	negative	negative	negative	negative	negative	negative	negative	positive
**102J**	positive	negative	negative	positive	positive	positive	positive	positive	negative	negative	negative	negative	negative	negative	pos and neg	positive

Table S2: Summary of translocation involved *Igh* and *Igl* genes

Mouse ID	Junction#	Chr #: Coordinate	Left region gene Involved	Chr #: Coordinate	Right region gene Involved
46J	1	Chr12:114664185	within Sμ	Chr15:61816455	Upstream of 1st exon of c-myc
46J	2	Chr12:114666343	Between JH4 and Eμ enhancer	Chr15:61815808	Upstream of 1st exon of c-myc
46J	3	Chr12:114576833	within Sγ1 region	Chr15:61816454	Upstream of 1st exon of c-myc
46J	5	Chr16:19260300	149bp dns of Vλ2 of Igλ locus	ChrX:69535637	15539 bp at 5' side: gamma-aminobutyric acid (GABA-A) receptor and 88925 bp at 3' side: melanoma-associated antigen 10
46J	6	Chr12:114793947	Between Igh-V7183 and J_H locus	Chr17:77509610	1559676 bp at 5' side: protein FAM98A and 1098678 bp at 3' side: cysteine-rich motor neuron 1 protein precursor
46J	33	Chr6:69318142	Igκ locus, in Vκ cluster	Chr8:75696092	ND
46J	38	Chr12 116464670	Upstream of Gm9227, between IghV8-6 and Ighv1-54	Chr2:157854651	Rprd1b
46J	39	Chr12:116852685	Between Ighv1-68 and Gm5660	Chr1:82561682	ND
119J	9	Chr12:114662370	within Sμ of Igh locus	Chr15:62002785	Pvt1 locus
119J	10	Chr12:114499669	within Sα of Igh locus	Chr15:62002643	Pvt1 locus
125J	8	Chr16:19085149	Vλ1 exon of Igλ locus	Chr7:139481912	ND
125J	30	Chr12:114892049	5' upstream of Igh-VQ52	Chr9:3486677	ND
125J	31	Chr12:114663535	within Sμ region	Chr15:61815797	Upstream of 1st exon of c-myc
125J	32	Chr12:117054332	Upstream of Gm18341-pseduo gene and Ighv8-14	Chr18:70277322	ND
125J	42	Chr12:114658536	3' of Cμ exon 4	Chr15:61810202	Upstream of 1st exon of c-myc
196J	7	Chr12:114666491	Between J_{H4} and Eμ enhancer	Chr15:61816078	Upstream of 1st exon of c-myc
196J	28	Chr12:114662359	within Sμ of Igh locus	Chr15:61816960	Upstream of 1st exon of c-myc
196J	29	Chr12:117210092	Upstream of Ighv1-83	Chr7:20527635	ND
196J	43	Chr6:70148134	between V8-26 and V8-21 of Igκ locus	Chr19:27764973	261672 bp at 5' side: pumilio domain-containing protein KIAA0020 and 73308 bp at 3' side: transcription factor RFX3
202J	27	Chr12:114631865	Upstream of Sγ3	Chr15:61816673	Upstream of 1st exon of c-myc

Table S3: Validation of Translocations by PCR, Sanger Sequencing or FISH

Junction	Mouse ID	Allele frequency	PCR	Sequence	FISH
Junction 1	46J	0.33	Validated	Validated	Validated
Junction 2	46J	0.26	Validated	Validated	Validated
Junction 3	46J	0.75	Validated	Validated	Validated
Junction 5	46J	0.30	Validated	Validated	ND
Junction 7	196J	0.67	Validated	Validated	Validated
Junction 8	125J	0.78	Validated	Validated	ND
Junction 9	119J	0.83	Validated	Validated	Validated
Junction 10	119J	0.82	Validated	Validated	Validated
Junction 27	202J	0.75	ND	ND	Validated
Junction 28	196J	0.67	ND	ND	Validated
Junction 31	125J	0.38	ND	ND	Validated
